# Mental Health Diagnoses in Patients With Mycosis Fungoides and Potential Impact on Oncologic Outcomes

**DOI:** 10.1002/cam4.70577

**Published:** 2025-03-07

**Authors:** Jackson N. Howell, Bayarmaa Mark, David A. Wada, Marianne Bowling, Mei Wei, Shane L. Lloyd, David K. Gaffney, Amit Maity, Michael J. LaRiviere, William G. Rule, Jon D. Grant, Vikrant Deshmukh, Michael Newman, Ankita Date, Mia Hashibe, Randa Tao

**Affiliations:** ^1^ Huntsman Cancer Institute Salt Lake City Utah USA; ^2^ Department of Radiation Oncology University of Utah School of Medicine Salt Lake City Utah USA; ^3^ Department of Dermatology University of Utah School of Medicine Salt Lake City Utah USA; ^4^ Department of Hematology/Oncology University of Utah School of Medicine Salt Lake City Utah USA; ^5^ Department of Radiation Oncology University of Pennsylvania Philadelphia Pennsylvania USA; ^6^ Department of Radiation Oncology Mayo Clinic Phoenix Arizona USA; ^7^ Department of Radiation Oncology Intermountain Health Salt Lake City Utah USA; ^8^ Utah Population Database University of Utah Salt Lake City Utah USA; ^9^ Division of Public Health, Department of Family and Preventive Medicine University of Utah Salt Lake City Utah USA

**Keywords:** hematology, mental health, mycosis fungoides, supportive care

## Abstract

**Background:**

We investigated mental health diagnoses (MHDs) in mycosis fungoides (MF) patients compared to the general population, evaluated risk factors, and studied survival outcomes in a large population database.

**Methods:**

MF patients from the Utah Cancer Registry diagnosed from 2001 to 2014 were matched with up to five general population individuals from the Utah Population Database. MHDs were retrospectively tracked in both populations (median follow‐up = 6.67 years). Risk factors for new MHDs among MF patients were studied using the Cox proportional hazards model. Overall survival (OS) and disease‐specific survival (DSS) were assessed using Kaplan–Meier analysis.

**Results:**

The incidence of anxiety disorders (HR = 1.99, 95% CI [1.16, 3.42]) and delirium/dementia disorders (HR = 2.43, 95% CI [1.05, 5.63]) was higher among MF patients than the matched general population. Among MF patients, Charlson Comorbidity Index (CCI) ≥ 2 and BMI < 18 kg/m^2^ were risk factors for new anxiety disorders. Radiation therapy, CCI ≥ 2, and female gender were risk factors for new delirium/dementia disorders. The 15‐year OS was worse for MF patients with versus without an MHD (36% vs. 81%, HR 2.62, 95%CI [1.24, 5.65]). The 15‐year DSS also worsened for MF patients with versus without an MHD (63% vs. 97%, HR 6.55, 95%CI [1.64, 26.2]).

**Conclusions:**

MF patients developed anxiety and delirium/dementia disorders at rates above the general population, and MHDs correlated with worse DSS and OS. Careful mental health monitoring may be an actionable step towards improving health‐related quality of life in this population.

## 1 Introduction

The predominant histological subtype of primary cutaneous T‐cell lymphoma (CTCL) is mycosis fungoides (MF), with an incidence of approximately 4 in 1,000,000 [1]. The prevalence is much higher due to the extended disease course. Except in rare cases, MF is not curable, and patients are often treated with a series of skin‐directed and systemic therapies as well as radiation therapy to prevent disease progression and palliate symptoms [2, 3, 4].

MF is considered an indolent malignancy, with Stage IA patients having a median overall survival (OS) of 35.5 years and a 20‐year risk of disease progression of 18% [5]. However, the rate of disease progression typically accelerates with stage advancement, and there is a significant reduction in median OS between patients with stage IIA (15.8 years) and stage IIB (4.7 years) disease, the latter of which is clinically marked by the development of cutaneous tumors [5]. Over the past 20 years, investigators have explored various psychosocial and mental health effects of MF and its treatment. Because of the prolonged course of this disease and its many symptomatic, cosmetic, and functional impacts on daily life, attention has recently been given to using patient‐centered quality of life (QoL) evaluations to guide treatments. This attention to QoL is particularly pertinent to the MF population, where prior research has suggested that patients experience an increased incidence of depressive and anxiety disorders [6, 7, 8, 9, 10, 11, 12]. We aimed to investigate these findings in our regional population, identify risk factors for developing mental health diagnoses, and evaluate if mental health diagnoses impacted patient survival.

## 2 Methods

We performed a retrospective cohort analysis of MF patients and the general population using the Utah Population Database. We identified patients by querying the Utah Cancer Registry for new MF diagnoses between 2001 and 2014. Each MF patient was matched with up to five cancer‐free individuals in the general population based on birth year, birth state, sex, and date of last residence in Utah. The Utah Population Database is a unique tool that links records from the University of Utah Health and Intermountain Healthcare (the two largest medical providers in the state, caring for the majority of residents) with the Utah Cancer Registry (a National Cancer Institute Surveillance, Epidemiology, and End Results database) and also incorporates family history records, residential histories, the Social Security Death Index, and certificates of birth, marriage, and death from the state of Utah. Mental health diagnoses were identified by analysis of the *International Classification of Diseases* [*9th Revision*] (ICD‐9) codes available within these databases. This study was approved by the University of Utah's Resource for Genetic and Epidemiologic Research and its Institutional Review Board.

The *χ* ^2^ test (*α* = 0.05) was used to compare baseline characteristics between the MF and general population cohorts. Follow‐up in the MF cohort was calculated as the time from initial MF diagnosis to the date of last follow‐up or death. Follow‐up in the general population cohort was calculated as the time from the index date (date of the matched MF patient's initial cancer diagnosis) to the date of last follow‐up or death. During follow‐up, the time from the index date to the documentation of any new mental health diagnoses (incident diagnoses) was recorded alongside the total number of individuals with a documented mental health diagnosis in their medical record before the cancer diagnosis date or index date (the prevalent diagnoses). The Cox proportional hazards model was used to compare the risk of developing incident mental health diagnoses during the follow‐up period between the two cohorts.

The Cox proportional hazards model was also used to investigate factors among MF patients associated with incident mental health diagnoses. Such factors included age, sex, disease stage, treatment interventions (e.g., surgery, radiation therapy, chemotherapy), BMI (based on driver's license records), and the Charlson Comorbidity Index (CCI) at baseline. The CCI is a validated index assessing a patient's comorbid disease burden, assigning weighted points for 16 designated comorbid conditions [13, 14].

Kaplan–Meier survival analysis was also performed, including the entire MF and general population cohorts, focusing on overall survival (OS) and disease‐specific survival (DSS). Log‐rank testing and hazard ratios were utilized to compare survival outcomes. In the survival analyses, patients were analyzed by the presence of MF and mental health diagnoses (both pre‐existing and incident during follow‐up).

To maintain patient confidentiality per the rules of the Department of Health and Human Services, data that could identify ≤ 10 individuals were not enumerated in the tables or text of this manuscript.

## 3 Results

A total of 124 MF patients met study criteria, and 545 individuals without MF from the general population were then identified and matched. The baseline characteristics of the two groups are presented in Table 1. The MF patients had a higher baseline CCI compared to the general population. Differences in the prevalence of pre‐existing mental health diagnoses were not statistically significant between the MF patients and the general population. Median follow‐up was shorter for the MF cohort (6.67 years) than the general population cohort (8.09 years, *p* < 0.0001). Table 2 shows the demographic and available treatment information for the MF patients.

**TABLE 1:** Demographics of the mycosis fungoides patients and general population cohort.

	MF patients (*n* = 124)		General population (*n* = 545)		*p*
	*N*	%	*N*	%	
Sex					
Female	41	33.1	173	31.7	0.7758
Male	83	66.9	372	68.3	
Race					
White	117	94.4	494	90.6	0.1004
Black	^a^	^a^	^a^	^a^	
Native American	^a^	^a^	^a^	^a^	
Asian and Pacific Islander	^a^	^a^	17	3.1	
More than one race	^a^	^a^	10	1.8	
Missing	^a^	^a^	17	3.1	
Hispanic ethnicity					
Non‐Hispanic	105	84.7	386	70.8	**< 0.0001**
Hispanic	^a^	^a^	42	7.7	
Missing	^a^	^a^	117	21.5	
Birth year					
1919–1940	31	25.0	132	24.2	0.9986
1941–1950	27	21.8	112	20.6	
1951–1960	32	25.8	144	26.4	
1961–1970	16	12.9	71	13.0	
1971–1980	^a^	^a^	59	10.8	
1981–1986	^a^	^a^ ^y^	27	5.0	
Baseline BMI					
≤ 24.9	51	41.1	216	39.6	0.5624
25–29.9	55	44.4	227	41.7	
≥ 30	18	14.5	102	18.7	
Baseline CCI					
0	69	55.6	381	69.9	**0.0045**
1	30	24.2	103	18.9	
≥ 2	25	20.2	61	11.2	
Pre‐existing MHD					
Yes	42	33.9	140	25.7	0.0646
No	82	66.1	405	74.3	
Follow‐up (years)					
≤ 5	46	37.1	144	26.4	**< 0.0001**
5+	78	62.9	401	73.6	
Median	6.67		8.09		

Abbreviations: BMI, body mass index; CCI, Charlson comorbidity index; MF, mycosis fungoides; MHD, mental health diagnosis.

^a^ Data that could identify ≤ 10 individuals have not been enumerated to protect patient confidentiality. The bold values were significant of *p* < 0.05.

**TABLE 2:** Clinical characteristics of the patients with mycosis fungoides.

	*N*	%
Diagnosis year		
2001–2005	46	37.1
2006–2010	46	37.1
2011–2014	32	25.8
Age at Diagnosis		
19–39	23	18.5
40–59	53	42.7
60–87	48	38.7
Stage		
Local	103	83.1
Regional or advanced	21	16.9
Primary site		
Skin of head	^a^	^a^
Skin of upper limb and shoulder	^a^	^a^
Skin of trunk	28	22.6
Skin of lower limb and hip	14	11.3
Overlapping lesion of skin	^a^	^a^
Skin, NOS	50	40.3
Missing	12	9.7
Surgery		
No	70	56.5
Yes	42	33.9
Missing	12	9.7
Radiation		
No	99	79.8
Yes	13	10.5
Missing	12	9.7
Chemotherapy		
No	92	74.2
Yes	20	16.1
Missing	12	9.7

^a^ Data that could identify ≤ 10 individuals have not been enumerated to protect patient confidentiality.

**FIGURE 1:**
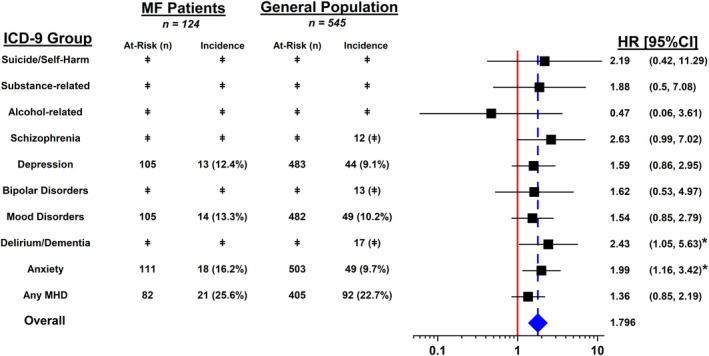
Incidence of mental health diagnoses (MHDs) among patients with mycosis fungoides (MF) and the general population. *95% confidence interval not inclusive of 1; ǂData that could identify ≤ 10 individuals have not been enumerated to protect patient confidentiality.

The incidence of new anxiety disorders (HR [95% CI], 1.99 [1.16, 3.42]) and delirium/dementia disorders (2.43 [1.05, 5.63]) was significantly higher in the MF patients compared to the general population (Figure 1). Schizophrenia and related psychotic disorders trended positively in the MF group but were ultimately not statistically significant (2.63 [0.99, 7.02]). The results of the subsequent risk factor analysis are presented in Table 3. A baseline CCI ≥ 2 (3.54 [1.23, 10.13]) and BMI < 18 kg/m^2^ (10.55 [1.23, 89.73]) were significantly associated with the development of anxiety disorders in the MF cohort. Notably, there was no correlation between incident anxiety disorders and sex. Risk factors associated with incident delirium/dementia disorders in the MF cohort included a baseline CCI ≥ 2 (7.88 [1.53, 40.66]), treatment with radiation therapy (5.08, [1.12, 23.0]), and female sex (4.85 [1.15, 20.52]).

**TABLE 3:** Risk factor analysis for incident mental health diagnoses identified in the patients with mycosis fungoides.

	Anxiety			Delirium, dementia, amnestic, and other cognitive disorders		
	All mycosis fungoides patients (*N* = 124)					
	*N*	%	HR (95% CI)	*N*	%	HR (95% CI)
Surgery						
No	^a^	^a^	Reference	^a^	^a^	Reference
Yes	^a^	^a^	1.62 (0.57, 4.62)	^a^	^a^	4.39 (0.85, 22.68)
Chemotherapy						
No	12	14	Reference	^a^	^a^	Reference
Yes	^a^	^a^	1.05 (0.23, 4.69)	0	—	—
Radiation						
No	11	12.5	Reference	^a^	^a^	Reference
Yes	^a^	^a^	1.63 (0.45, 5.86)	^a^	^a^	**5.08 (1.12, 23.05)**
Stage						
Localized	14	15.4	Reference	^a^	^a^	Reference
Regional	^a^	^a^	1.58 (0.52, 4.80)	0	—	**—**
Advanced	0	—	**—**	0	—	**—**
Charlson Comorbidity Index						
0	^a^	^a^	Reference	^a^	^a^	Reference
1	^a^	^a^	1.22 (0.36, 4.16)	^a^	^a^	0.91 (0.08, 10.12)
2+	^a^	^a^	**3.54 (1.24, 10.13)**	^a^	^a^	**7.88 (1.53, 40.66)**
Baseline BMI						
< 18 kg/m^2^	^a^	^a^	**10.55 (1.24, 89.73)**	0	—	—
18–24.9 kg/m^2^	^a^	^a^	Reference	^a^	^a^	Reference
< 25–29.9 kg/m^2^	^a^	^a^	0.47 (0.16, 1.40)	^a^	^a^	0.61 (0.14, 2.71)
> 30 kg/m^2^	^a^	^a^	0.90 (0.24, 3.32)	^a^	^a^	0.62 (0.07, 5.55)
Sex						
Male	12	15.8	Reference	^a^	^a^	Reference
Female	^a^	^a^	1.19 (0.44, 3.17)	^a^	^a^	**4.85 (1.15, 20.52)**

Abbreviation: BMI, body mass index.

^a^ Data that could identify ≤ 10 individuals have not been enumerated to protect patient confidentiality. The bold values were significant of *p* < 0.05.

**FIGURE 2:**
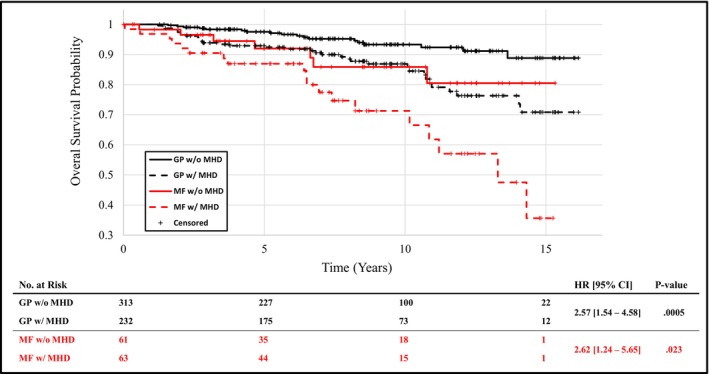
Overall survival of the mycosis fungoides (MF) and general population (GP) cohorts stratified by MF and mental health diagnosis (MHD).

**FIGURE 3:**
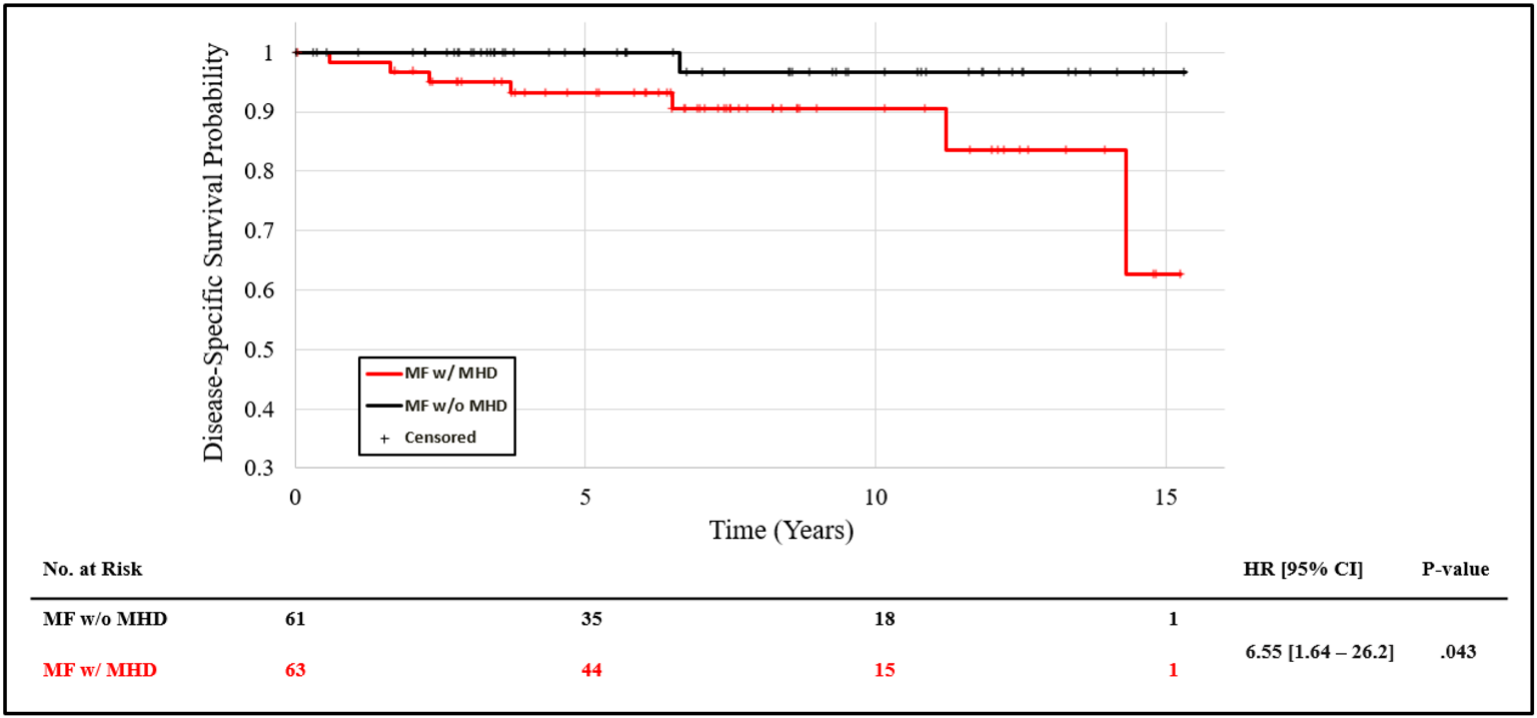
Disease‐specific survival of the mycosis fungoides (MF) cohort stratified by mental health diagnosis (MHD).

In both the MF (2.62 [1.24, 5.65]) and general population (2.57 [1.54–4.58]) cohorts, OS was shorter for individuals with versus without a mental health diagnosis documented at any time (Figure 2). In the MF cohort, the presence of a mental health diagnosis also significantly correlated with reduced DSS (Figure 3, 6.55 [1.64, 26.2]). The 5‐, 10‐, and 15‐year OS rates for MF patients without a mental health diagnosis were 92%, 86%, and 81% versus 87%, 71%, and 36% with a mental health diagnosis (Figure 2). The 5‐, 10‐, and 15‐year DSS rates were 100%, 97%, and 97% in MF patients without a mental health diagnosis versus 93%, 91%, and 63% for patients with such a diagnosis (Figure 3).

## 4 Discussion/Conclusions

Patients with MF had an increased risk of being diagnosed with anxiety and delirium/dementia disorders compared to the general population. The presence of mental health diagnoses, either pre‐existing or new, was also significantly correlated with worse OS and DSS. Our risk factor analysis identified CCI ≥ 2 and BMI < 18 as being significantly associated with the development of incident anxiety disorders in the MF cohort. Additionally, CCI ≥ 2, treatment with radiation therapy, and female gender were identified as risk factors for the development of incident delirium/dementia disorders.

MF is a potentially morbid disease with significant intrusions into daily life related to skin erythema and pruritus. Demierre and colleagues surveyed the membership of the Mycosis Fungoides Foundation in 2005, finding that patients' MF symptoms had a profound impact on their global functioning and social well‐being [15]. In the survey results, patients noted that their skin lesions and pruritis affected their choice of clothing, subjective feelings of attractiveness, and sexual function. Approximately 66% of patients reported fatigue that hindered their job performance, interactions with loved ones, and daily non‐work activities such as exercise. Each of these domains has a major impact on patient quality of life, and several meta‐analyses have linked consistent exercise with better quality of life among cancer patients [16, 17]. A study of patients with cutaneous anaplastic large cell lymphoma additionally showed that consistent physical activity improves quality of life in patients with cutaneous lymphomas [18]. Other studies have shown that MF patients face an increase in depressive and anxiety disorders during their illness, often with a greater weight of psychiatric illness arising in female patients [6, 7, 8, 9, 10, 11, 12, 15]. For instance, Sampogna and colleagues performed a detailed evaluation of health‐related quality of life (HRQoL) for 95 cutaneous lymphoma patients at their institution using the Skindex‐29 and EORTC QLQ‐C30 evaluation tools. They further observed that female patients reported worse physical disability, HRQoL, pain, and psychological impairment, particularly depressive and anxiety disorders, than the male respondents with the same severity of disease. Additionally, Engin and colleagues, in their survey of 52 Turkish patients with MF, noted increasing levels of depression with worse disease stages and for patients diagnosed later in life.

Notable differences between our results and prior literature are that sex did not arise as a significant risk factor in the development of anxiety disorders, nor did depressive disorders present at a higher rate than in the general population. Multiple factors may contribute to these differences, including regional, religious, socioeconomic, and racial differences in the observation and acceptance of mental health diagnoses. Our MF cohort was predominantly white, and only one‐third were female. This is different from the MF population nationally where African American patients develop the disease at an equivalent or higher rate than Caucasians [19, 20].

We observed a significantly decreased OS among MF patients with mental health diagnoses. One explanation for this could be that a worse burden of disease could have negative impacts on both mental health and survival. For instance, more advanced stages and the worsening of disease have previously been tied to rates of depression in this population [11]. However, this may not be the only explanation. Our data did not demonstrate increased rates of depression in MF patients compared to the general population, nor did it find that patients with more advanced disease stages had an increased risk for anxiety or delirium/dementia disorders.

Treatment with radiation therapy was identified as a risk factor for the development of delirium/dementia disorders. However, it is challenging to tell what role age and additional treatments play as confounding variables. This correlation has not previously been reported in the literature and is unexpected after radiation therapy for MF because the treatment is directed at the skin. We believe this finding may be a chance finding in the small sample of patients that developed delirium/dementia disorders that may not be statistically significant in a larger sample.

We have previously examined mental health diagnoses and survival outcomes in other cancer patient populations using the Utah Population Database with similar findings. For instance, patients with Hodgkin lymphoma were found to be at higher risk of anxiety, depression, substance‐related disorders, suicide, and intentional self‐harm injuries compared to the general population [21]. Colorectal and prostate cancer patients were found to be at high risk of developing mental health diagnoses, particularly depressive disorders, and cancer patients with a mental health diagnosis had worse OS compared to cancer patients without a mental health diagnosis [21, 22, 23].

Our study has several notable limitations. First, our ability to monitor mental health diagnoses using diagnosis codes relies on the integrity of the electronic medical record, which may not always be accurate or complete. Alternatively, using diagnoses in the medical record reduces the possibility of recall bias and low response rates that may hinder survey‐based studies. Second, we expect that our data are impacted by surveillance bias, as MF patients would likely have had more significant interaction with the healthcare system than the general population, leading to greater sensitivity of mental health diagnosis detection. Third, because the patients were included from the Utah Population Database, our results may not be generalizable to all MF patients nationally, especially regarding race and ethnicity distributions, as fewer African American patients were represented in our database. Finally, we recognize that this population‐based study cannot account for several known and unknown confounding factors that could affect the diagnosis of a mental health disorder. Our results are hypothesis‐generating and we do not infer any causality in the medical literature outside of a well‐conducted randomized controlled trial. The rarity of MF makes it challenging to conduct well‐powered prospective studies to evaluate its mental health consequences. To this point, one strength of our study is its relatively large sample size for this rare disease. Another strength of this study is its population‐based design, which allows for comparisons with the general population through matching.

Our data suggest that MF patients in Utah are diagnosed with specific mental health disorders at rates above the general population and that mental health diagnoses may be associated with worse OS and DSS. The careful monitoring of mental health and providing multidisciplinary psychiatric and social resources, as appropriate, could improve HRQoL in this population. Mental health is likely one actionable factor among many that contribute to a complicated clinical milieu for MF patients, who face varied social and economic factors associated with chronic illness and long‐term cancer care [10]. Therefore, future studies should explore validating these findings nationally and investigating interventions to support mental health and improve quality of life in patients with MF.

## Précis

In a population study of Utah, patients with mycosis fungoides experienced some mental health conditions at higher rates than the general population. Mycosis fungoides patients with mental health conditions had worse survival outcomes than those without them.

## Author Contributions

**Jackson N. Howell:** project planning, data analysis, manuscript writing, manuscript editing. **Bayarmaa Mark:** data collection, data analysis. **David A. Wada:** manuscript editing. **Marianne Bowling:** manuscript editing. **Mei Wei:** manuscript editing. **Shane L. Lloyd:** manuscript editing. **David K. Gaffney:** manuscript editing. **Amit Maity:** manuscript editing. **Michael J. LaRiviere:** manuscript editing. **William G. Rule:** manuscript editing. **Jon D. Grant:** manuscript editing. **Vikrant Deshmukh:** data collection. **Michael Newman:** data collection. **Ankita Date:** data collection. **Mia Hashibe:** project planning, data analysis, manuscript editing. **Randa Tao:** project planning, data analysis, manuscript editing.

## Ethics Statement

This study was approved by the University of Utah's Resource for Genetic and Epidemiologic Research and its Institutional Review Board (IRB_00065816).

## Consent

A waiver for patient consent was granted by the University of Utah IRB/Ethics Committee as this project was performed using de‐identified data collected within the scope of a publicly funded research database.

## Conflicts of Interest

S.L.L. has received consulting fees from Cancer Study Group, expert witness fees from Kipp and Christian, and travel reimbursement from Naveris. M.J.L. has received drug‐only funding from Merck for a clinical trial that includes mycosis fungoides patients. M.W. holds consultant or advisory roles for AstraZeneca, Relay Therapeutics, Novartis, Seagen, and Merck. No other authors hold relevant conflicts of interest.

## Data Availability

Research data will be shared upon reasonable request to the corresponding author.
